# The chain mediating effect of self-efficacy and health literacy between proactive personality and health-promoting behaviors among Chinese college students

**DOI:** 10.1038/s41598-025-00936-0

**Published:** 2025-05-08

**Authors:** Shouying Wang, Junjun Wei, Panpan Zhang, Jie Song, Jilong Chen, Genqiang Li

**Affiliations:** 1 School of Public Health, Xinxiang Medical University, Xinxiang, 453003 China; 2 School of Health Management, Xinxiang Medical University, Xinxiang, 453003 China

**Keywords:** Health-promoting behavior, Proactive personality, Self-efficacy, Health literacy, College students, Psychology, Health care

## Abstract

Health-promoting behaviors are essential for college students as they develop lifelong health habits. To investigate how to cultivate health-promoting behaviors among college students, this study aimed to investigate the influence of proactive personality on health-promoting behaviors and to explore the mediating roles of self-efficacy and health literacy through a cross-sectional study. A total of 664 college students from six colleges in Xinxiang, China, were conveniently sampled to complete questionnaires, including the Proactive Personality Scale, General Self-Efficacy Scale, 12-item Short-Form Health Literacy Scale, and Short-Form Health Promotion Scale. Path analysis indicated that proactive personality was directly associated with health-promoting behaviors (effect value: 0.146). The mediating roles of self-efficacy (effect value: 0.165) and health literacy (effect value: 0.080) were significant. A chain mediating effect of self-efficacy and health literacy was also observed (effect value: 0.028). The positive effect of proactive personality (β = 0.146, *P* < 0.001), self-efficacy (β = 0.421, *P* < 0.001) and health literacy (β = 0.234, *P* < 0.001) on health-promoting behaviors was significant. These findings suggest that self-efficacy and health literacy play a chain mediating role between proactive personality and health-promoting behavior. Future interventions should target proactive personality, self-efficacy, and health literacy to enhance health-promoting behaviors in college students.

## Introduction

In the context of increasing concerns about adolescent health, a growing body of research has focused on identifying risk factors and potential interventions to address health challenges among student populations. Evidence suggests that over half of Chinese students are in suboptimal health, a reversible transitional state between health and disease, which highlights the urgent need for effective strategies to promote healthy behaviors and improve health outcomes^1^. In recent years, many studies have explored ways to remain healthy^2^ (for example, physical exercise^3^), prevent diseases^4^ (for example, psychological and behavioral issues^5–7^), change unhealthy behavior^8,9^ (for example, internet addiction^10^) and promote health^11^ (for example, individual psychology and behavior^12,13^). Even minor improvements in health behaviors can yield substantial benefits for health outcomes^14^. Proactive health has emerged as an innovative approach to proactively improving college students’ health^15^. It encompasses all social activities focused on health and prevention, integrating health goals into all policies and spanning the entire population and life cycle^16^. As the primary responsible party for their health, college students should take a proactive approach to actively promoting their health status.

Health-promoting behavior was defined as those behaviors in which individuals engage to enhance their health and well-being by Pender^17^. Some studies suggest that individuals with a proactive personality tend to exhibit more positive behaviors^18^ and better health outcomes^19^, making it a critical factor in shaping health-promoting behaviors. A time-related personality was significantly associated with health-promoting behaviors among college students, and one factor in this personality could positively predict health-promoting behaviors^20^. Many studies have sought to understand the mechanisms shaping health-promoting behavior using theories and developing evidence-based interventions^21,22^. For example, the Theory of Planned Behavior (TPB) has been used to predict mental health-promoting behaviors among young adults^23^. Pender’s Health Promotion Model (HPM) emphasizes that health-promoting behaviors are influenced not by a single factor, but rather by the combined effects of individual characteristics, behavior-specific cognitions, and environmental factors^17^. Moreover, Social Cognitive Theory (SCT) identifies self-efficacy as a belief in one’s capability to take a series of actions to reach a certain goal and as an important determinant of motivation, affect, and action^24^. This theory emphasizes the importance of self-efficacy in behavior. Self-efficacy was an important positive predictive factor of health-promoting behaviors^25^. Another study found that health literacy was positively associated with health-promoting behaviors and concluded that health literacy was vital for health-promoting behaviors, the higher the health literacy, the more likely an individual will be to exhibit health-promoting behaviors^26^. Despite the growing body of research on health-promoting behaviors, significant gaps remain in understanding the mechanisms underlying these behaviors. Based on Pender’s HPM and previous studies, we hypothesize that personality, self-efficacy, and health literacy are important factors influencing health-promoting behaviors with certain connections and mechanisms among them. Although previous studies have identified a range of factors associated with health-promoting behaviors, they have seldom explored the chain-mediated effects of multiple mediating variables, such as self-efficacy and health literacy. Furthermore, limited attention has been given to the role of proactive personality as a significant trait in shaping health-related behaviors, underscoring the need for a more nuanced understanding of how personality traits interact with other mediating factors. To address these limitations, this study builds on Pender’s HPM, integrating self-efficacy and health literacy to develop a comprehensive theoretical framework. Methodologically, a chain mediation model is employed to simultaneously examine the mediating roles of self-efficacy and health literacy, Additionally, by incorporating proactive personality as a key predictor, the study broadens the scope of factors influencing health-promoting behaviors and offers new insights into the interplay between personality traits and health behaviors. Finally, the synergistic effects of health literacy and self-efficacy are highlighted, providing a foundation for innovative health education and intervention strategies.

## Theoretical basis and hypothesis

### Pender’s health promotion model

HPM was developed by Pender. It is widely recognized in health sciences for its ability to explain and predict health-promoting behaviors. The model emphasizes the multidimensional nature of health-promoting behaviors, highlighting the interplay between individual characteristics, behavior-specific cognitions, and socio-environmental factors^17^. Specifically, individual characteristics are categorized into biological, psychological, and sociocultural dimensions, while behavior-specific cognition factors include perceived benefits, perceived barriers, self-efficacy, activity-related affect, and various interpersonal and situational influences. Our study aligns closely with this model by integrating self-efficacy and health literacy as key mediating variables, which are central to the HPM’s conceptualization of behavior-specific cognitions. Specifically, Self-efficacy reflects an individual’s confidence in their ability to perform health-promoting behaviors, a critical component of the HPM’s “perceived self-efficacy” construct. Health literacy represents an individual’s capacity to access, understand, and apply health-related information, which aligns with the HPM’s emphasis on behavior-specific cognitions and affect. By situating our study within the HPM, we provide a unified theoretical structure that not only justifies the inclusion of these mediators but also explains their sequential roles in linking proactive personality to health-promoting behaviors. In our study, self-efficacy serves as a proximal mediator, translating the motivational influence of proactive personality into actionable health behaviors. This aligns with the HPM’s assertion that self-efficacy is a critical driver of behavior change. Health literacy, as a distal mediator, bridges the gap between self-efficacy and health-promoting behaviors by enabling individuals to effectively utilize health information and resources. This is consistent with the HPM’s focus on “behavioral outcomes” and the role of cognitive factors in shaping health behaviors. Our study extends the HPM by introducing a chain mediation model, which elucidates the sequential pathways through which proactive personality influences health-promoting behaviors. Although the TPB^27^ and SCT^28^ have significantly contributed to predicting health behaviors, they primarily emphasize specific factors, such as behavioral intentions or self-efficacy, while potentially overlooking other influential elements. In contrast, HPM provides a multidimensional framework that highlights the combined influence of individual characteristics, behavior-specific cognitions, and environmental factors on health behaviors. HPM not only integrates core concepts like self-efficacy but also aligns closely with health literacy through its emphasis on behavioral cognitions. The multidimensional and comprehensive nature of HPM makes it the most suitable theoretical foundation for this study. What’s more, individual characteristics and experiences directly influence health-promoting behaviors or indirectly affect them through behavior-specific cognitions and affective factors^17^. Therefore, HPM provides a strong theoretical foundation that supports and validates our research hypotheses.

### The relationship between proactive personality and health-promoting behavior

Bateman defined the concept of a proactive personality as a relatively stable tendency to affect environmental change^29^. Individuals with more proactive personalities engage in more proactive behaviors^30^. Pender’s HPM indicated that personal psychological factors, such as self-esteem, self-motivation, and perceived health status, influence health behavior^17^. Self-motivation is closely related to proactive personality and can be seen as one of its manifestations^31^. Given the above reasoning, proactive personality can be classified as a psychological factor within Pender’s HPM. A person with a highly proactive personality may adopt health-promoting behaviors and remain healthy^32,33^. Therefore, our study theorizes that a proactive personality is positively correlated with health-promoting behaviors (Hypothesis 1).

### The mediating role of self-efficacy

Self-efficacy refers to one’s beliefs about accomplishing a task and can influence the choice of activities, effort, persistence, and achievement^34^. It is the foundation of human motivation and action^24^. Self-efficacy is a significant predictor of health promotion activities^35^. Another study also reported that self-efficacy was an independent predictor of the total health-promoting lifestyle^36^. Therefore, self-efficacy is an important factor in health-promoting behavior, and finding the factors that enhance self-efficacy has the potential to improve health-promoting behaviors. In the aspect of motivation, self-efficacy and personality share common ground. However, in Pender’s HPM, proactive personality acts as a personal psychological factor, and self-efficacy functions as a behavior-specific cognition and affect factor that contributes to health-promoting behaviors. Proactive personality has been shown to significantly correlate with and predict self-efficacy^37,38^. Therefore, we can speculate that individuals with a proactive personality attach greater importance to the exertion of self-determination and autonomous abilities which will further strengthen their self-efficacy. In conclusion, individuals who have proactive personality are more likely to have a high level of self-efficacy, and self-efficacy can predict health-promoting behaviors.

### The mediating role of health literacy

Health literacy is an individual’s knowledge, motivation, and competencies to access, understand, appraise, and apply health information to make judgments and decisions in daily life concerning healthcare, disease prevention, and health promotion to maintain or improve the quality of life^39^. Health literacy is critical in empowering individuals to make decisions about personal health and enabling their engagement in collective health-promoting actions to address the determinants of health^40^. According to the definition of health literacy, it provides essential cognitive support in identifying the benefits and barriers of action. Perceived benefits and perceived barriers are important factors of behavior-specific cognitions and affect section in HPM^17^. Incorporating health literacy into the behavior-specific cognitions and affect section of the model is highly appropriate. Therefore, proactive personality and health literacy are important factors in health-promoting behaviors. One person with proactive personality has more motivation to engage in learning lots of health-related knowledge and health-promoting skills, which represents that they will have a high level of health literacy and then be prone to adopting health-promoting behaviors. From the perspective of SCT, health literacy serves as the cognitive basis for individuals in health-promoting behaviors^41^. Individuals with higher health literacy can better understand the importance and applicability of health information, improve their ability to utilize health services, and thus are more likely to adopt health-promoting behaviors. There are also some studies consistent with the above opinion. Health literacy was a positive predictor of health-promoting behaviors^26,42^. Moreover, positive aspects of personality were shown to be positively correlated with health literacy^43,44^. Therefore, health literacy may play a mediation role in personality and health-promoting behaviors.

### Chain mediation of self-efficacy and health literacy

**Fig. 1:**
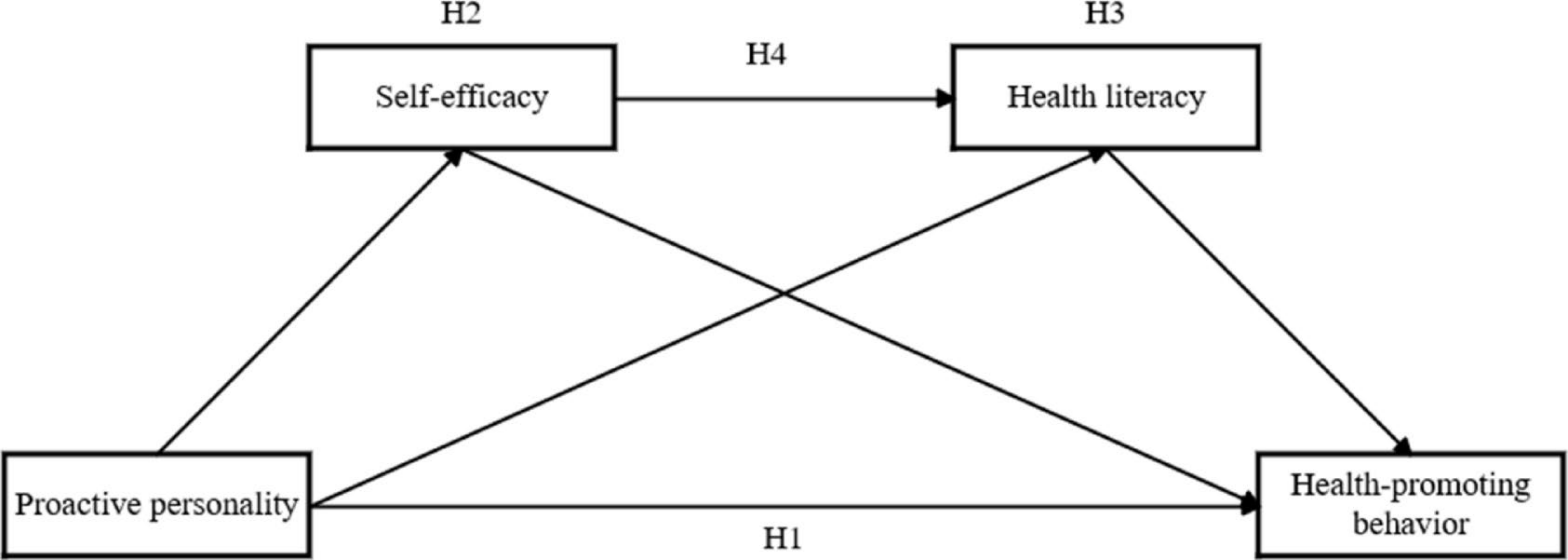
Chain mediation hypothesis model.

As stated above, while proactive personality acts as a personal psychological factor, self-efficacy and health literacy jointly act as behavior-specific cognitions and affect factors of Pender’s HPM. Consequently, we can speculate that self-efficacy and health literacy play important roles in the relationship between proactive personality and health-promoting behavior. Moreover, self-efficacy is associated with health literacy to some extent^45^. From the perspective of self-efficacy predicts health literacy, self-efficacy is defined as an individual’s speculation and judgment regarding their capacity to accomplish a particular behavior^24^. Individuals possessing high self-efficacy are more strongly impelled to obtain health knowledge, heighten health awareness, and are more inclined to endeavor and acquire proficiency in health skills. Several studies have demonstrated that self-efficacy was a strong predictor of health literacy^46,47^. Consequently, self-efficacy emerges as an antecedent variable of health literacy, influencing the attainment of health knowledge, the development of health awareness, and the mastery of health skills. Based on these studies and previous hypotheses, this study posits that self-efficacy and health literacy may play chain-mediating roles in proactive personality and health-promoting behaviors. Our study aims to explore college students’ inner motivation to improve health-promoting behaviors, so it is more suitable to place self-efficacy as an antecedent variable before health literacy. Therefore, it is valuable to incorporate self-efficacy and health literacy into the mediating variable and reveal their effects on proactive personality and health-promoting behaviors among college students (see Fig. 1).

In conclusion, this study has four purposes. First, it examined whether proactive personality had a significant positive correlation with health-promoting behaviors. Second, this study explored whether self-efficacy played a mediating role in the relationship between proactive personality and health-promoting behaviors. Third, the study examined whether health literacy played a mediating role in the relationship between proactive personality and health-promoting behaviors. Finally, the present study explored whether self-efficacy and health literacy played a chain mediating role in the relationship between proactive personality and health-promoting behaviors among college students.

Thus, this study proposes the following hypotheses:

#### Hypothesis 1

Proactive personality is positively correlated with health-promoting behaviors among college students.

#### Hypothesis 2

Self-efficacy among college students plays a mediating role in the relationship between proactive personality and health-promoting behaviors.

#### Hypothesis 3

Health literacy among college students plays a mediating role in the relationship between proactive personality and health-promoting behaviors.

#### Hypothesis 4

Self-efficacy and health literacy among college students play a chain mediating role in the relationship between proactive personality and health-promoting behaviors.

### Participants and methods

#### Participants

This study, which used a cross-sectional survey research design and convenience sampling method, has passed the ethical review to investigate college students from six colleges in Xinxiang, China. The data of the research was obtained through an open survey between 14 December 2023 and 31 December 2023 with informed consent. Undergraduate students aged 17 to 26 who possess the ability to complete the questionnaire independently and have basic self-care skills to manage their health behaviors participated voluntarily in an online e-questionnaire survey whose items included were in alternate order. In our survey, demographic and key study variables were set as mandatory to ensure data completeness and minimize missing values, so there is no missing data. Moreover, participants who have finished the questionnaire will obtain a pen as a gift. A total of 753 college students were investigated, and 664 valid questionnaires were retained after excluding invalid responses according to the exclusion criteria: (1) excessively short completion times; (2) clear patterns of repetitive answer choices; (3) unreliable answers of demographic characteristics. The study was approved by the institution’s ethics review committee of the Xinxiang Medical University [XYLL-20230335]. To determine the appropriate sample size for this study, a power analysis was conducted using G*Power 3.1.9.7^48^. The medium effect size (f²) of 0.15 was selected, the significance level (α) was set at 0.05, and the desired statistical power (1-β) was set at 0.95. Given that the most complex regression equation in the mediation model included three predictors, the analysis determined that a minimum sample size of 119 participants was required. Due to the limited representativeness of the sample resulting from convenience sampling, we increased the anticipated sample size by 30%, resulting in a minimum required sample size of approximately 155 participants.

#### Description analysis

Table 1 shows the demographic characteristics of participants. The participants were divided into two groups based on the age threshold of 20 years aligning with the typical progression of university education. Body Mass Index (BMI) was classified according to the Working Group on Obesity in China (WGOC) criteria: underweight (< 18.5 kg/m²), normal weight (18.5–23.9 kg/m²), overweight (24.0–27.9 kg/m²) and obesity (≥ 28.0 kg/m²)^49^. Among the valid questionnaires, 392 (59%) were female while 272 (41%) were male. Of these respondents, 392 (59%) were 20 or under 20 years of age, and 272 (41%) were older than 20 years. The majority of the college students (648, 97.6%) were of Han nationality. A total of 101 (15.2%) participants were medical students. In terms of the academic year, 146 (22%) were freshmen; 171 (25.8%) were sophomores; 161 (24.2%) were juniors; 160 (24.1%) were seniors; and 26 (3.9%) were fifth-year medical students. Regarding BMI, 104 (15.7%) participants were classified as overweight or obese. 529 (79.7%) participants considered their father’s health status above general health condition, as well as 501 (75.4%) of their mother’s health status. Only 96 (14.5%) participants consider them under average rich. A total of 469 (70.6%) college students considered themselves relatively healthy or very healthy. Nearly all participants (651, 98%) reported having received school-based health education.

**Table 1:** Demographic characteristics of the participants (*N* = 664).

Demographic Variables	Groups	*N*	Composition Ratio (%)
Gender	Male	272	41.0
	Female	392	59.0
Age	≤ 20	392	59.0
	> 20	272	41.0
Nationality	Han nationality	648	97.6
	Minority nationality	16	2.4
Major	Medical	101	15.2
	Non-medical	563	84.8
Grade	Freshmen	146	22.0
	Sophomore	171	25.8
	Junior	161	24.2
	Senior	160	24.1
	Fifth year student	26	3.9
BMI	Thin	119	17.9
	Normal	441	66.4
	Overweight	86	13.0
	Fat	18	2.7
Father’s health status	Died	11	1.6
	Very unhealthy	0	0.0
	Relatively unhealthy	18	2.7
	General health condition	106	16.0
	Relatively unhealthy	278	41.9
	Very healthy	251	37.8
Mother’s health status	Died	5	0.8
	Very unhealthy	2	0.3
	Relatively unhealthy	29	4.4
	General health condition	127	19.1
	Relatively unhealthy	252	37.9
	Very healthy	249	37.5
Family’s financial situation	Very difficult	9	1.3
	relatively poor	96	14.5
	Average	463	69.7
	relatively good	75	11.3
	very good	21	3.2
Self-reported health status	Very unhealthy	2	0.3
	Relatively unhealthy	16	2.4
	General health condition	177	26.7
	Relatively healthy	303	45.6
	Very healthy	166	25.0
School health education	Never	13	2.0
	Seldom	93	14.0
	Sometimes	314	47.3
	Often	179	26.9
	Usually	65	9.8

### Measuring tools

#### Proactive personality scale

The proactive personality scale was developed by Bateman^29^ and revised into a Chinese version by Shang Jiayin et al.^50^, and its validity and reliability have been proven to be acceptable. The Chinese version scale contains 11 items. The scale questions were set as declarative sentences. For example, “If I see others in trouble, I will do my best to help.” “I am good at turning problems into opportunities.” “I am always looking for better ways to act.” Each item was scored on a 7-point Likert scale, ranging from 1 to 7 points. The scores of the 11 items were summed, ranging from 7 to 77 points. The higher total scores indicate more proactive personality. Cronbach’s alpha for the proactive personality scale in this study was 0.942.

#### General self-efficacy scale

The General Self-Efficacy Scale (GSES) was developed by Ralf Schwarzer^51^ and later translated and revised by Zhang Jianxin et al.^52^. It includes 10 items. For example, “If I try hard enough, I can always solve difficult problems.” “If someone opposes me, I can find ways and means to get what I want.” “It is easy for me to stick to my ideals and achieve my goals.” The questionnaire items were scored on a 4-point Likert scale ranging from 1 (absolutely wrong) to 4 (absolutely right). The scores of the 10 items were summed, ranging from 10 to 40 points. The higher total scores indicate higher general self-efficacy. Cronbach’s alpha for the GSES was 0.944. The reliability index of the scale is well-established, indicating that it can be applied to the Chinese population.

### 12-Item Short-Form health literacy scale (HLS-SF12)

The 12-item Short-Form Health Literacy Scale (HLS-SF12) was developed based on the European Health Literacy Survey Questionnaire (HLS-EU-Q), which was created by Kristine Sørensen et al.^53^. Building upon the HLS-EU-Q, Duong et al. later designed the 12-item short form of the Health Literacy Scale^54^. It measures public health literacy and includes three dimensions. The three dimensions are healthcare, disease prevention, and health promotion. There are 12 questions on this scale. For example, “How easy would you find information on treatments of illnesses that concern you?” “How easy would you understand the leaflets that come with your medicine?” “How easy would you judge the advantages and disadvantages of different treatment options?” Each item is rated on a 4-point Likert scale ranging from “very difficult” to “very easy.” The scores of the 12 items were summed, ranging from 12 to 48 points. The higher total scores indicate higher health literacy. Cronbach’s alpha for the health literacy scale was 0.903. The reliability index of the scale was well-established.

### The short-form version of the adolescent health promotion scale (AHP-SF)

The short-form version of the Adolescent Health Promotion Scale (AHP-SF) was developed based on the original Adolescent Health Promotion Scale (AHP), which was created by Chen Meiyen et al. in 2003^55^. Building upon the AHP scale, Chen Meiyen et al. later designed the AHP-SF scale in 2014^56^. It comprises six dimensions: nutrition, social support, health responsibility, life appreciation, exercise, and stress management. There are 21 questions on this scale. For example, “I eat three meals daily.” “I choose foods without too much oil.” “I eat breakfast daily.” Each item is rated on a 5-point Likert scale (1 = never, 2 = sometimes, 3 = often, 4 = usually, and 5 = always). The scores of the 21 items were summed, ranging from 21 to 105 points. The higher total scores indicate better health-promoting behaviors. Cronbach’s alpha for all items in this scale was 0.940, indicating that this scale had good reliability.

### Data analysis

Harman’s single-factor test was applied to examine common method bias. Descriptive analysis, Pearson correlation analysis, comparative analysis, linear regression analysis of the multiple mediation models and chain mediation analysis were conducted in SPSS (version 27.0). Categorical variables were presented as frequencies and percentages. Continuous variables were described as either mean ± standard deviation (Mean ± SD) or median with interquartile range (Q1-Q3). Control variables were selected through a two-step process. First, comparative analyses identified variables with statistically significant effects on health-promoting behaviors. Comparisons between two groups were performed using independent samples t-tests. For comparisons involving three or more groups, one-way analysis of variance (ANOVA) was applied. When the assumptions of normality and homogeneity of variances were violated, the Kruskal-Wallis test served as the non-parametric alternative. Additionally, gender^57^ and BMI^58^ were associated with health-promoting behavior and included as control variables. Model 6 of the SPSS PROCESS macro was employed to examine the relationship between proactive personality and health-promoting behavior, focusing on the chain mediating effects of self-efficacy and health literacy. We conducted 5000 bootstrap resampling iterations to assess model fit and estimate 95% confidence intervals (95% CI). The significance level of the statistical results was set at *P* < 0.05. A significant effect was indicated if the bootstrap 95% CI did not include zero.

## Results

### Common method bias test

To examine potential common method bias, an unrotated exploratory factor analysis was performed using Harman’s single-factor test^59^. The results revealed 10 common factors with eigenvalues greater than one. The first factor accounted for 32.31% of the variance, which is below the commonly accepted threshold of 40%. Thus, there was no significant common method bias detected in this study.

### Correlation analysis

As shown in Table 2, Pearson correlation analysis was conducted to examine the relationships among proactive personality, self-efficacy, health literacy and health-promoting behavior. The health-promoting behavior of college students was significantly and positively correlated with their proactive personality (*r* = 0.464), self-efficacy (*r* = 0.621), and health literacy (*r* = 0.534). College students’ proactive personalities were significantly and positively correlated with self-efficacy (*r* = 0.427) and health literacy (*r* = 0.504), and their self-efficacy and health literacy were also significantly and positively correlated with each other (*r* = 0.480). These findings provide strong evidence to support Hypothesis 1, laying the foundation for subsequent regression analysis and the development of a chain mediation model.

**Table 2:** Statistics of pearson correlation coefficient.

Variables	M ± SD	1	2	3	4
Proactive personality	59.743 ± 11.081	1			
Self-efficacy	27.586 ± 5.778	0.427^***^	1		
Health literacy	36.233 ± 5.659	0.504^***^	0.480^***^	1	
Health-promoting behavior	74.276 ± 13.059	0.464^***^	0.621^***^	0.534^***^	1

Notes: *** *P* < 0.001.

### Regression analysis of proactive personality, self-efficacy, health literacy, and health-promoting behaviors

This study employed regression analysis to explore the potential mediating effects of self-efficacy and health literacy in the relationship between proactive personality and health-promoting behaviors among college students. In Table 3, the result of comparative analyses indicated that major, father’s health status, mother’s health status, family’s financial status, self-reported health, and school health education were significantly associated with health-promoting behavior. Gender and BMI were also controlled according to their potential influence. Therefore, major, father’s health status, mother’s health status, family’s financial status, self-reported health, school health education, gender and BMI were included as covariates. The results of regression analysis (see Table 4) revealed that proactive personality (β = 0.146, *P* < 0.001), self-efficacy (β = 0.421, *P* < 0.001), and health literacy (β = 0.234, *P* < 0.001) positively predicted health-promoting behaviors. Additionally, proactive personality had a significant positive predictive effect on self-efficacy (β = 0.392, *P* < 0.001) and health literacy (β = 0.343, *P* < 0.001). Self-efficacy also demonstrated a significant positive predictive effect on health literacy (β = 0.304, *P* < 0.001). In addition, the analysis revealed that proactive personality explained 27.1% of the variance in health-promoting behavior and 22.8% of the variance in self-efficacy. Moreover, proactive personality and self-efficacy together explained 36.6% of the variance in health literacy, while proactive personality, self-efficacy, and health literacy collectively explained 49.3% of the variance in health-promoting behavior.

**Table 3:** Comparative analyses on health-promoting behavior.

Demographic Variables	Groups	*N* (%)	Health-promoting behavior scores Mean ± SD / Median(Q1-Q3)	t/F/H	*P*
Gender	Male	272(41.0%)	73.952 ± 13.268	-0.531	0.595
	Female	392(59.0%)	74.500 ± 12.924		
Age	≤ 20	392(59.0%)	73.816 ± 12.936	-1.088	0.277
	> 20	272(41.0%)	74.938 ± 13.229		
Nationality	Han nationality	648(97.6%)	74.312 ± 13.066	0.453	0.650
	Minority nationality	16(2.4%)	72.813 ± 13.116		
Major	Medical	101(15.2%)	76.792 ± 12.477	2.109	0.035
	Non-medical	563(84.8%)	73.824 ± 13.120		
Grade	Freshmen	146(22.0%)	72.206 ± 13.768	1.941	0.102
	Sophomore	171(25.8%)	75.491 ± 11.904		
	Junior	161(24.2%)	74.093 ± 13.131		
	Senior	160(24.1%)	74.388 ± 13.628		
	Fifth year student	26(3.9%)	78.346 ± 11.060		
BMI	Thin	119(17.9%)	75.185 ± 13.245	0.393	0.758
	Normal	441(66.4%)	74.204 ± 12.705		
	Overweight	86(13.0%)	73.837 ± 15.120		
	Fat	18(2.7%)	72.111 ± 10.029		
Father’s health status	Died	11(1.6%)	67.000(54.500–80.500)	10.864	0.028
	Very unhealthy	0(0%)	0.000(0.000)		
	Relatively unhealthy	18(2.7%)	68.000(57.000–81.000)		
	General health condition	106(16.0%)	73.000(64.000–83.000)		
	Relatively unhealthy	278(41.9%)	74.000(65.000–84.000)		
	Very healthy	251(37.8%)	77.000(65.000–84.000)		
Mother’s health status	Died	5(0.8%)	72.000(66.000–73.000)	26.559	< 0.001
	Very unhealthy	2(0.3%)	65.000(63.000–67.000)		
	Relatively unhealthy	29(4.4%)	66.000(63.000–78.000)		
	General health condition	127(19.1%)	71.000(62.500–79.000)		
	Relatively unhealthy	252(37.9%)	73.000(65.000–84.000)		
	Very healthy	249(37.5%)	79.000(66.000–84.000)		
Family’s financial situation	Very difficult	9(1.3%)	67.000(58.000–75.000)	10.710	0.030
	relatively poor	96(14.5%)	74.000(63.000–84.000)		
	Average	463(69.7%)	74.000(65.000–84.000)		
	relatively good	75(11.3%)	79.000(68.000–84.000)		
	very good	21(3.2%)	83.000(74.000–85.000)		
Self-reported health status	Very unhealthy	2(0.3%)	61.000(59.000–63.000)	46.595	< 0.001
	Relatively unhealthy	16(2.4%)	63.500(54.500–67.000)		
	General health condition	177(26.7%)	70.000(63.000–81.000)		
	Relatively healthy	303(45.6%)	75.000(66.000–84.000)		
	Very healthy	166(25.0%)	79.500(67.000–84.000)		
School health education	Never	13(2.0%)	70.000(63.000–73.000)	36.569	< 0.001
	Seldom	93(14.0%)	68.000(62.000–77.000)		
	Sometimes	314(47.3%)	74.000(63.000–84.000)		
	Often	179(26.9%)	77.000(68.500–84.000)		
	Usually	65(9.8%)	84.000(70.000–84.000)		

**Table 4:** Regression analysis of the multiple mediation model.

	Health-promoting behavior			Self-efficacy			Health literacy			Health-promoting behavior		
	β	SE	*t*	β	SE	*t*	β	SE	*t*	β	SE	*t*
Gender	0.054	0.072	0.748	-0.103	0.074	-1.389	0.044	0.067	0.657	0.094	0.060	1.565
Major	-0.116	0.094	-1.234	-0.159	0.097	-1.633	-0.356	0.088	-4.032***	0.045	0.080	0.563
BMI	-0.085	0.053	-1.594	-0.006	0.055	-0.116	-0.030	0.050	-0.595	-0.075	0.045	-1.683
Father’s health status	-0.014	0.045	-0.302	-0.028	0.046	-0.602	-0.019	0.042	-0.452	0.005	0.038	0.122
Mother’s health status	0.046	0.047	0.980	0.115	0.049	2.364*	0.027	0.044	0.601	-0.017	0.040	-0.418
Family’s financial situation	0.064	0.054	1.172	0.093	0.056	1.650	-0.084	0.051	-1.659	0.038	0.046	0.833
Self-reported health status	0.152	0.050	3.031**	0.069	0.052	1.345	0.103	0.047	2.202*	0.094	0.042	2.225*
School health education	0.137	0.040	3.431***	0.084	0.041	2.040*	0.040	0.037	1.066	0.086	0.033	2.580*
Proactive personality	0.420	0.034	12.229***	0.392	0.035	11.090***	0.343	0.035	9.826***	0.146	0.034	4.372***
Self-efficacy							0.304	0.035	8.588***	0.421	0.033	12.591***
Health literacy										0.234	0.035	6.690***
R^2^	0.271			0.228			0.366			0.493		
F	26.980***			21.434***			37.758***			57.661***		

Notes: *P < 0.05, **P < 0.01, ***P < 0.001.

### Mediating effects of self-efficacy and health literacy in the relationship between proactive personality and health-promoting behaviors

**Fig. 2:**
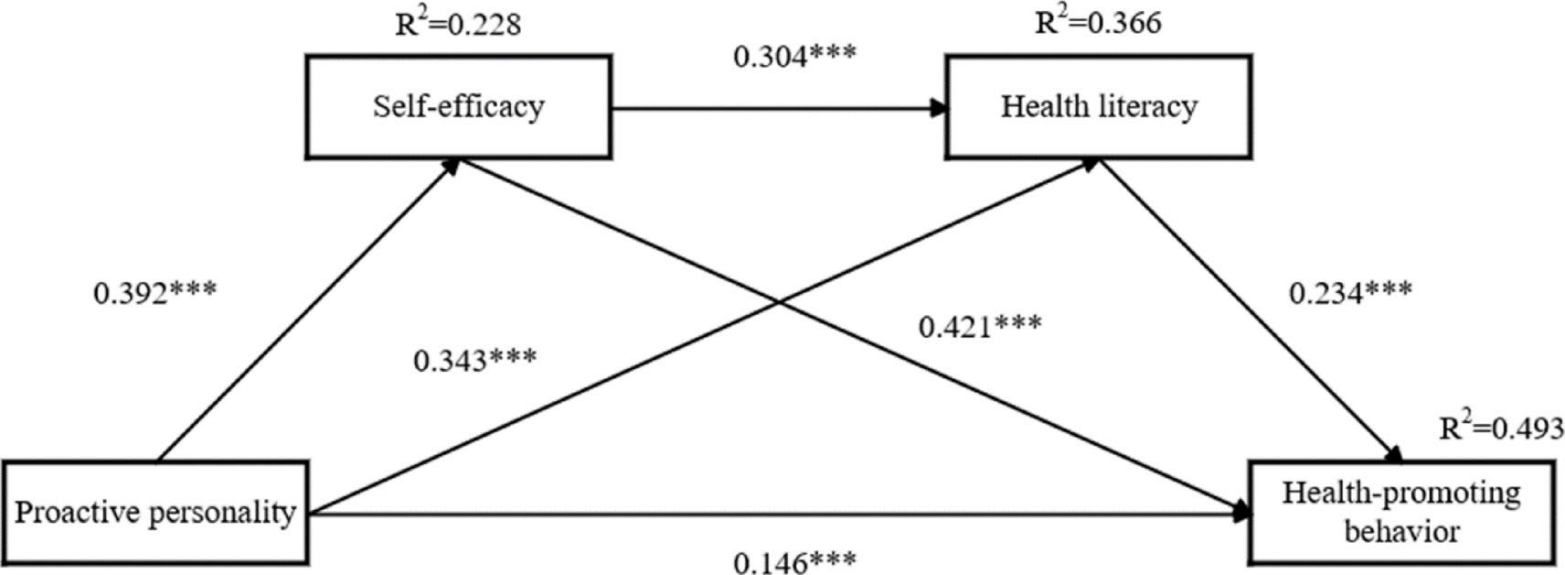
The final model and model path coefficient. Notes: *P < 0.05, **P < 0.01, ***P < 0.001.

SPSS was used to test Hypotheses 2, 3, and 4, examining the mediating effects of self-efficacy and health literacy on the relationship between proactive personality and health-promoting behaviors. Major, father’s health status, mother’s health status, family’s financial status, self-reported health, school health education, gender and BMI were treated as covariates in this analysis. The results of the model path coefficients highlight four significant paths (see Fig. 2). The bootstrap method was applied to test the mediating effects (see Table 5). The direct effects revealed that proactive personality was positively associated with health-promoting behaviors (effect size = 0.146, 95% CI [0.081, 0.212]). As the confidence interval did not include zero, it confirmed a significant effect of proactive personality on health-promoting behaviors, with an effect value of 0.146 and a direct effect contribution of 34.76%. In the PP-SE-HPB path (effect size = 0.165, 95% CI [0.119, 0.214]), self-efficacy had a significant mediating effect (39.29%), with an effect value of 0.165. In the PP-HL-HPB path (effect size = 0.08, 95% CI [0.048, 0.117]), health literacy demonstrated a significant mediating effect (19.05%), with an effect value of 0.08. Finally, in the PP-SE-HL-HPB path (effect size = 0.028, 95% CI [0.016, 0.042]), self-efficacy and health literacy jointly exhibited a significant mediating effect (6.67%), with an effect value of 0.028. Comparing the mediating effects revealed that the chain mediating effect value was significantly smaller than the independent mediating effects of both self-efficacy (95% CI [0.094, 0.186]) and health literacy (95% CI [0.026, 0.084]). Moreover, the independent mediating effect of health literacy was significantly smaller than that of self-efficacy (95% CI [0.026, 0.147]).

**Table 5:** Test results of bootstrap mediation effect.

Type of Effect	Effect size	Boot SE	Boot LLCI	Boot ULCI	Ratio effect
Direct Effect	0.146	0.034	0.081	0.212	34.76%
Total Mediation Effect	0.274	0.032	0.213	0.339	65.24%
Total Effect	0.42	0.034	0.353	0.488	100.00%
PP→SE→HPB	0.165	0.024	0.119	0.214	39.29%
PP→HL→HPB	0.08	0.017	0.048	0.117	19.05%
PP→SE→HL→HPB	0.028	0.007	0.016	0.042	6.67%
C1	0.085	0.031	0.026	0.147	
C2	0.137	0.023	0.094	0.186	
C3	0.052	0.015	0.026	0.084	

Abbreviations: PP, proactive personality; SE, self-efficacy; HL, health literacy; HPB, health-promoting behavior.

## Discussion

To better understand how proactive personality influences health-promoting behaviors, this study developed a chain mediation model incorporating self-efficacy and health literacy. By testing this model, the study reveals the psychological mechanisms underlying this relationship and provides practical implications for promoting health-related behaviors among college students.

### Proactive personality and health-promoting behaviors

 The results of this study confirm Hypothesis 1. Proactive personality significantly predicts health-promoting behavior. Personality traits have been shown to significantly predict health-promoting behaviors^60^. College students with high levels of proactive personality are more likely to face adversity with resilience, take effective measures, and adapt to the environment, all of which benefit their mental health^61^. Proactive students often engage in positive activities^33^, leading to achievements^62^ that further motivate health-promoting behaviors. Beyond prior findings that personality traits can predict health-related behaviors, this study extends the understanding by emphasizing how proactive personality fosters a stronger intrinsic motivation for engaging in health-promoting behaviors. Proactive individuals tend to actively seek opportunities for self-improvement, and develop goal-directed behaviors despite challenges^63^. These individuals may also possess a greater tendency to seek health-related information and proactively implement lifestyle adjustments, which explains why they engage more in health-promoting behaviors. Moreover, according to the Self-Determination Theory, individuals with a proactive personality are more likely to experience autonomous motivation, which drives consistent health-related actions^64^. Thus, rather than merely predicting health behaviors, proactive personality may function as a key initiator of self-driven health engagement. Future research could explore whether this effect persists across different populations and contexts.

### Mediating role of self-efficacy

 The findings support Hypothesis 2, demonstrating that self-efficacy mediates the relationship between proactive personality and health-promoting behaviors among college students. Beyond confirming the general association between self-efficacy and health behaviors, this study highlights the role of self-efficacy as a motivational bridge. Self-efficacy represents an individual’s confidence and competence in completing tasks and has consistently been identified as a positive predictor of health-promoting behaviors^65,66^. According to the SCT, individuals with high self-efficacy are more likely to perceive health behaviors as beneficial and achievable, which increases their commitment to adopting them^24^. Thus, self-efficacy functions as a key motivator for health-promoting behaviors^67^. The identification of antecedents that enhance self-efficacy emerges as a crucial factor in facilitating health-promoting behaviors. Current studies have demonstrated that proactive personality positively correlates with self-efficacy and serves as its predictor^37^. Individuals with a proactive personality are prone to adopt health-promoting behaviors^68^. Similar studies consistent with our results have shown that self-efficacy mediates the relationship between personality traits and health-promoting behaviors^60^. Therefore, individuals with proactive personalities are more likely to possess self-efficacy, which serves as a motivational driver for adopting health-promoting behaviors.

### Mediating role of health literacy

 This study validates Hypothesis 3, establishing that health literacy mediates the relationship between proactive personality and health-promoting behaviors. Health literacy enables individuals to access, evaluate, and utilize health-related information effectively, guiding them toward better health decisions^69^. Numerous studies align with this finding, highlighting the positive correlation between health literacy and health-promoting behaviors^70,71^. While a study has reported insignificant correlations, this could stem from varying economic conditions and cultural backgrounds^25^. Moreover, a significant positive correlation was found between proactive personality and health literacy, and previous studies were also consistent with our result^43,44^. The observed positive correlation suggests that students with proactive personalities are more inclined to acquire, evaluate, and apply health-related information, which facilitates better health decisions. Compared to previous studies, our findings reveal the potential of proactive personality in enhancing health-promoting behaviors through improved health literacy, thereby advocating individuals to harness their proactive nature to actively develop health literacy and apply health-related knowledge and skills in daily life to maintain a healthy lifestyle. Additionally, they may have a stronger tendency to engage in self-directed learning, further enhancing their health literacy. This underscores the importance of integrating proactive personality development into health education programs to maximize their impact.

### The Chain mediating effect of self-efficacy and health literacy between proactive personality and health-promoting behaviors

The results verify Hypothesis 4, demonstrating that self-efficacy and health literacy play a chain mediating role in the relationship between proactive personality and health-promoting behaviors. This suggests that proactive individuals first develop higher self-efficacy, which in turn enhances their health literacy, ultimately leading to better health behaviors. Studies showed that self-efficacy strongly predicts health literacy^46^, and both factors positively influence health-promoting behaviors^72^. Previous research has shown that psychological resources, such as optimism, significantly mediate the impact of health-related concerns on well-being^73^. This suggests that individuals with a proactive personality, who tend to possess stronger psychological resources, are more inclined to engage in health-promoting behaviors. Compared to previous studies, our research incorporates both psychological and cognitive factors to investigate the pathways of cultivating health-promoting behaviors, which enables a comprehensive revelation of the underlying behavioral psychological mechanisms and provides a crucial research foundation for subsequent interventions. In HPM, proactive personality was recognized as an individual character, whereas self-efficacy and health literacy were added as behavior-specific cognition factors to refine the theoretical model. Our study extends this understanding by demonstrating that self-efficacy and health literacy function as mediators between proactive personality and health-promoting behavior, highlighting the crucial role of both psychological and cognitive resources in fostering health-promoting behaviors. When proactive individuals develop strong self-efficacy, they are more likely to seek out and comprehend health-related knowledge, leading to more informed health choices. This pathway provides a theoretical foundation for designing interventions that simultaneously enhance self-efficacy and health literacy to improve health behaviors. Our study is the first one to fully investigate the mediating role of self-efficacy and health literacy in the relationship between proactive personality and health-promoting behavior. Therefore, the strategic cultivation and utilization of proactive personality traits significantly enhance the development of self-efficacy and health literacy, thereby establishing a robust psychological foundation that promotes the consistent adoption and maintenance of health-promoting behaviors at the individual level.

### Influence of demographic characteristics on health-promoting behaviors

 This study used comparative analyses to test the difference in health-promoting behaviors in terms of demographic characteristics (see Table 3). Results showed that major, father’s health status, mother’s health status, family’s financial status, self-reported health status, and school health education had significant differences in health-promoting behaviors. In particular, college students majoring in medicine, better parents’ health statuses, better self-reported health status and more school health education experiences performed better in health-promoting behavior. College students are in a pivotal phase of establishing lifelong lifestyle habits. Grounded in SCT, the health-related behaviors exhibited by their immediate environment and social networks can exert a subtle yet significant influence on their engagement in health-promoting activities. Consequently, it is imperative to provide college students with enhanced access to health promotion resources through targeted family-based and school-based health education programs. Such initiatives can bolster their self-efficacy in managing personal health, thereby fostering a synergistic interplay between individual agency and societal support in health promotion efforts. This integrated approach is essential for optimizing the efficacy of health promotion interventions and achieving sustainable behavioral outcomes.

## Limitations and future directions

 This study has several limitations. First, this study employed a convenience sampling method, which may limit the representativeness of the sample. To enhance sample representativeness in future research, cluster sampling or simple random sampling could be considered as alternative approaches. Second, reliance on self-reported data introduces the possibility of social desirability bias. Therefore, future studies should adopt more objective measures, such as using a health management App to record the data on health-related behaviors. Third, the study’s focus on college students limits the generalizability of the findings to other populations. Future research should explore diverse populations, including working professionals and older adults, to examine whether the observed mechanisms hold across different life stages. Our study primarily focuses on promoting health at the individual level among university students. In the future, a broader perspective could be explored to develop effective strategies that foster a collaborative approach to health promotion, integrating both individual and societal efforts. Furthermore, developing intervention programs based on this model and testing their effectiveness would offer valuable scientific evidence for health promotion practices.

## Conclusions

 This study demonstrates that proactive personality directly influences health-promoting behavior and indirectly affects it through the chain-mediating roles of self-efficacy and health literacy. Specifically, four pathways were identified: (1) Proactive personality significantly predicts health-promoting behaviors. (2) Proactive personality indirectly influences health-promoting behaviors through self-efficacy. (3) Proactive personality indirectly influences health-promoting behaviors through health literacy. (4) Self-efficacy and health literacy jointly play a chain mediating role in the relationship between proactive personality and health-promoting behaviors. By fostering proactive personality, self-efficacy, and health literacy, college students can develop more health-promoting behaviors. These findings provide valuable insights for future research and health promotion strategies.

## Data Availability

The datasets analyzed in the current study are available from the corresponding author upon request.
